# Vertebral Artery Injury with Coinciding Unstable Cervical Spine Trauma: Mechanisms, Evidence-based Management, and Treatment Options

**DOI:** 10.7759/cureus.7225

**Published:** 2020-03-09

**Authors:** Sarah Merrill, William Clifton, Fidel Valero-Moreno, Aaron Damon, Gazanfar Rahmathulla

**Affiliations:** 1 Neurological Surgery, Mayo Clinic Alix School of Medicine, Scottsdale, USA; 2 Neurological Surgery, Mayo Clinic, Jacksonville, USA; 3 Neurological Surgery, University of Florida College of Medicine, Gainesville, USA

**Keywords:** vertebral artery, cervical spine fracture, vertebral artery injury, anticoagulation, cervical spine trauma, vertebral artery dissection, traumatic injury, blunt cervical trauma, anatomy, cerebrovascular injury

## Abstract

Unstable traumatic cervical spine fracture is a commonly encountered neurosurgical issue. Concomitant vertebral artery injuries present a challenge in surgical decision-making regarding the timing and order of surgical intervention with respect to endovascular intervention and internal fixation of the unstable fracture. Currently, there are no studies that have specifically examined stroke rate or outcomes for patients who have vertebral artery injuries and unstable cervical spine fractures with respect to temporal treatment course. The purpose of this paper is to review the current evidence for the standards of diagnosis and management of vertebral artery injuries with coinciding unstable cervical spine injuries and propose an evidence-based algorithm for workup and treatment.

## Introduction and background

Blunt trauma of the cervical spine most commonly occurs in patients after high-speed motor vehicle collisions or falls. The incidence of vertebral artery injury (VAI) associated with cervical spine fractures is reported as high as 30% [1,2]. The treatment of traumatic VAIs depends on the extent of the vessel injury and patient symptoms [3,4]. In cases with concurrent unstable cervical spine trauma, the timing and sequence of surgical intervention for both the vessel injury and unstable fracture can be complicated. There is a paucity of high-level evidence in the literature regarding treatment algorithms for timing of anticoagulation and/or catheter-based intervention and cervical spine fixation in cases of VAI with concomitant unstable cervical spine injury. The purpose of this article is to review the current literature regarding the treatment of VAI and simultaneous cervical spine injury in order to assist providers with treatment decision-making.

## Review

The VA is conventionally divided into four anatomic segments. Segment I originates from the branch of the subclavian artery to its intersection with the vertebral transverse foramina of either C6 or C7 in most cases [5]. Segment II travels through the subaxial transverse foramina to C2. Segment III courses posterolaterally in a tortuous course from the transverse foramen of C2 to the posterior arch of C1, which renders it particularly vulnerable to stretch and rotational injuries [6,7]. Segment IV crosses the dura at the foramen magnum and runs superiorly to meet the proximal basilar artery. Approximately 15% of patients have a unilateral hypoplastic VA that may not be able to provide sufficient collateral flow if the contralateral artery is injured [8]. Although only a small percentage of acute traumatic VAIs present with neurologic deficits, delayed presentations from missed diagnoses have severe consequences if ischemic or embolic phenomenon occurs [9-11]. Neurologic manifestations of traumatic VAI are uncommon [12-14]. Vertebrobasilar ischemia may result in quadriparesis or sensory loss that may go unrecognized with simultaneous spinal cord injury [15,16]. Other symptoms from lateral medullary compromise such as cranial nerve palsies and sympathetic dysfunction may also occur. Unilateral VAI is rarely associated with neurologic symptoms due to extensive collateral flow from the contralateral VA as well as the anastomotic arterial network in the cervical musculature [17,18]. 

Pathophysiology of traumatic VAI

Traumatic VAIs stem from a variety of injuries to the bones and ligaments of the cervical spine, including fracture, subluxation/dislocation, excessive flexion and extension, and rotational injuries. Multiple types of fractures to the cervical vertebrae increase the risk of VAI. Fractures involving the transverse foramen have been associated with up to an 88% rate of VAI [5,6]. Unstable vertebral body fractures including tear-drop fracture (extending from the anterior vertebral body to the inferior endplate) and any fracture with a 3-mm anterolisthesis or greater are more likely to contribute to VAI [19]. A recent study of 435 patients with traumatic injury to the head and neck found a significant positive association of VAI with the presence of neurologic signs and vertebral fracture [18]. However, it is important to note that absence of fracture on radiologic studies does not exclude VAI.

Certain blunt forces to the head or cervical spine may result in movement of vertebrae relative to their native position. These dislocations or subluxations may be described in terms of the resulting positions of the facet joints. The mildest form of displacement involves a partially subluxed facet joint in which there is incomplete uncovering of the inferior facet. This may be accompanied by anterolisthesis and/or rotation of the vertebral body. A perched facet joint occurs when the inferior articular process of one vertebral process sits directly on the ipsilateral superior articular process of the vertebra below. If the superior vertebra subluxes anteriorly beyond this perched position, it becomes a ‘jumped’ facet joint. The superior facet becomes locked in place and may entrap the VA segment at the involvement level, leading to dissection or occlusion. This happens most often at the C4-C5 and C5-C6 levels [5,20].

Excessive shearing forces occurring during extreme flexion and extension, such as in a whiplash injury with rapid acceleration and deceleration, have been suggested as causes of intimal injuries in the cervical arteries [9,21,22]. In this case, the entrapment of the VA within the transverse foramina creates torque in opposing directions throughout the inciting event. This may occur with or without fracture. 

Injuries involving traumatic rotation of the cervical vertebrae include atlantoaxial rotatory fixation (presenting with an inability to return the head to anatomical baseline), traumatic isolation of the articular pillar, and unilateral dislocation [23]. Severe injuries of this nature demonstrate a rotation of C1-C2 of greater than 45 degrees, or a widening of the atlanto-dens interval of greater than 3 mm [24]. In some blunt traumas involving rotational forces, these signs may be absent if a portion of the force is translated into a fracture of the vertebrae; thus, less displacement can be expected if an articular mass fracture is present.

Etiology and diagnosis of VAI in cervical spine fractures

The incidence of VAIs varies greatly among studies in the literature. Prospective studies have found an incidence of up to 75%, depending on the mechanism of the injury [12,25]. The most frequent precipitating event is motor vehicle accident at high velocity, defined as greater than 40 miles per hour [18]. Less commonly reported traumatic events include falls, strangulation, pedestrian and bicycle accidents, and case reports of injuries from roller coasters, chiropractic spinal adjustment, and severe vomiting [12,17,19,26]. Rarely, medical procedures requiring hyperextension of the neck, such as esophagogastroduodenoscopy, have been reported to result in VA dissection [27]. Significant VAI is more likely to occur in patients less than 65 years of age than in younger patients with the same injury severity score [7].

The gold standard of diagnosis of VAI is digital subtraction angiography (DSA); however, computed tomography angiogram (CTA) has largely replaced DSA in most centers because of its time requirements and invasive nature [28,29]. Magnetic resonance imaging/magnetic resonance angiography (MRI/MRA) is also a useful imaging tool, and may also be used to diagnose ligamentous injury and spinal cord damage concurrently [30,31]. Neither CTA nor MRI/MRA have the sensitivity or specificity of DSA in diagnosing VAI. Guidelines regarding the selection and use of imaging modality for this pathology have yet to be established. There is evidence that screening in trauma patients reduces the risk of ischemic events by early treatment; however, it does not appear to have an effect on overall outcomes [13,18,32].

Classification and treatment of traumatic VAI

Blunt cerebrovascular injury is classified according to the Denver Scale. Anticoagulation vs. antiplatelet therapy has been studied without a significant difference in stroke risk [4,33]. Managing these injuries in patients with polytrauma can be difficult, since the majority of the time any anticoagulation or antiplatelet medication is relatively contraindicated in patients with traumatic brain injury or other trauma of the extremities [3,34-36]. The exact efficacy of anticoagulation or antiplatelet therapy in VAI is unknown. It has been reported that up to 40% of patients with VAI experience cerebral ischemic events at the time of diagnosis before treatment can be started [37]. Antiplatelet or anticoagulation is less likely to be effective once an ischemic event occurs, and raises a risk of hemorrhagic conversion of the infarcted area.

The standardization of the use of advanced imaging for screening purposes in trauma patients varies between institutions. A large prospective study by Biffl et al. grouped vertebral and carotid artery injuries together and showed that Grade 1 and 2 patients should be followed by successive invasive imaging as the majority of patients required a change in management after the results of the follow-up imaging [4]. Over 50% of Grade 1 patients completely healed with anticoagulation and required no further therapy, while Grade 2 injuries progressed to pseudoaneurysm formation in over 40% of patients. 

Endovascular treatment methods may be employed when antiplatelet or anticoagulation methods are contraindicated [11,24,33]. Endovascular treatment of spontaneous VA dissections has been extensively studied, but not so in traumatic injuries. The choice of endovascular treatment depends on the extent of the injury and the clinical picture responsible for the patient’s symptoms. Vessel dissections with distal emboli and good collateral flow may be treated by vessel sacrifice, whereas stenting may be a preferred option in cases with poor collateral flow or bilateral vessel injury.

The natural history of traumatic VAI is that over 90% of stenotic dissections resolve within several months and over half of occluded vessels recanalize [24,26]. This trend toward spontaneous vessel healing has promoted a culture of conservative management of traumatic VAIs in the acute setting, as opposed to aggressive surgical correction. 

Concomitant treatment of unstable cervical spine injuries

While there is substantial literature on the treatment of traumatic VAI, there is not a clear guideline on the timing of cervical spine fixation in patients with unstable fractures and VAIs. This presents an unanswered question on the timing of anticoagulation and surgery for these patients requiring medical therapy for their arterial injury. In general, anticoagulation is not started until the unstable injury is treated. Surgical fixation of the cervical spine injury may be delayed due to patient condition or time of presentation, which would delay the timing of anticoagulation initiation. Additionally, the timing of postoperative anticoagulation has not been well studied with respect to postoperative hematoma incidence and other postoperative complications. The decision-making process in these circumstances can be challenging, especially in patients with high-grade spinal cord injuries. Unstable fractures involving the transverse foramen, facet dislocations, or rotational injuries have a higher probability of VAI and should be further evaluated with CTA. For Denver Grade 1 or 2 VAI, stabilizing the cervical injury and if indicated decompressing the spinal cord should be employed first, followed by aspirin or Plavix therapy as soon as possible postoperatively. For Denver Grade 3 or 4 injuries, if there is no evidence of an expanding pseudoaneurysm, active bleeding (Grade 5), or a posterior fossa stroke, proceeding with spinal fixation first may decrease the likelihood of further vascular injury. If there is evidence for any one of the previously mentioned complications of Grades 3, 4, or 5 VAI, then it must be up to a multidisciplinary team to decide on the timing of fixation vs. endovascular treatment on a case-by-case basis.

There is anecdotal evidence that stabilization of an unstable cervical spine injury is protective against further VAI [32,38,39]. In some cases, endovascular treatment of the VAI may need to precede spinal fixation in patients with expanding pseudoaneurysms or embolic occlusions with neurologic deficits. The timing of antiplatelet and/or anticoagulation initiation postsurgery has not been well studied, and currently is left to the decision of the treating neurosurgeon. Delaying medical therapy in patients with low-grade injuries is dangerous and has been shown to cause an increase in the rate of neurologic morbidity and mortality [39].

## Conclusions

VAIs are relatively common in patients with simultaneous cervical spine trauma. Involvement of the transverse foramen, distracting or rotational injuries, and high-grade subluxation have been shown to be risk factors. Screening protocols vary between institutions, and there is not currently a universal evidence-based practice standard. CT angiogram has largely replaced DSA as the preferred imaging modality. The treatment of arterial injuries in spine trauma patients is complex and is currently performed on a case-by-case basis. It is important to initiate medical therapy as soon as possible in patients with low-grade VAIs after surgical fixation of unstable fractures to avoid neurologic morbidity from ischemic or embolic complications; however, there is no standard of timing for initiation of medical therapy or understanding of risk factors for post-operative complications. It is also not known if patients with VAI and cervical fractures are at higher risk of neurologic complications after internal fixation due to delays in initiating anticoagulation due to surgery. There is a gap in the current literature in standardizing a screening and treatment algorithm for patients with concomitant unstable cervical spine and vertebral artery injuries, which will need to be investigated in order to provide practitioners with a tool for surgical decision-making in this patient population. 
